# Enhanced Recovery After Surgery Protocol for Lumbar Spinal Surgery With Regional Anesthesia: A Retrospective Review

**DOI:** 10.7759/cureus.18016

**Published:** 2021-09-16

**Authors:** Lakshmi N Kurnutala, Joshua E Dibble, Sudhakar Kinthala, Michelle A Tucci

**Affiliations:** 1 Anesthesiology and Perioperative Medicine, University of Mississippi Medical Center, Jackson, USA; 2 Anesthesiology, Singing River Health System, Ocean Springs, USA; 3 Anesthesiology, Guthrie Robert Packer Hospital, Sayre, USA; 4 Anesthesiology, University of Mississippi Medical Center, Jackson, USA

**Keywords:** length of stay, opioids use, regiona anesthesia, lumbar spine surgery, eras protocol

## Abstract

Background

In the USA, spinal fusion surgery incurs the highest hospital cost. Despite the recent advances in the application of enhanced recovery after surgery (ERAS) protocols in these surgeries, the efficacy of these protocols in improving the perioperative outcomes remains unclear. We conducted a retrospective review as a quality improvement (QI) project to analyze the efficacy of the ERAS protocol with intraoperative modified thoracolumbar interfascial plane (mTLIP) block to determine whether these interventions reduce the length of stay (LOS) and opioid requirements during the postoperative period.

Methods

Retrospective reviews of adult patients (>18 yrs) who underwent elective lumbar spinal fusion or laminectomy at our institute were reviewed. Patients were administered oral gabapentin and acetaminophen preoperatively. Prior to incision, an mTLIP block was performed using liposomal bupivacaine. Intraoperatively, ketamine, ketorolac, and tranexamic acid were administered. Postoperative, pain control was treated with scheduled acetaminophen, ketorolac, and low-dose ketamine infusion. Hydromorphone and oxycodone were administered for breakthrough pain. Patients who underwent a similar procedure without ERAS protocol were chosen as controls to assess the efficacy of ERAS protocol. Data pertaining to patient demographics, operative and perioperative use of analgesics, LOS, 90-day readmissions, and morbidity were collected. Patients who underwent laminectomy and spinal fusion surgery were analyzed separately

Results

A total of 65 patients were identified; laminectomy (n- 24), spinal fusion surgery (n-41). In the laminectomy patients, treatment group (n-12) and the control group (n-12). Treatment group receiving the ERAS protocol with the regional anesthesia via the mTLIP (n= 12) opioid requirement was reduced by 51.42% [P = 0.03], and LOS was reduced by 2.04 days [P = 0.01] [0.75 days vs. 2.79 days]). In the spinal fusion patients, treatment group (n-15) and control group (n-26). Treatment group receiving the ERAS protocol with the use of regional anesthesia via the mTLIP group (n= 15), opioid requirement was reduced by 38.33% [P = 0.04]. No difference in LOS was observed at 5.4 days vs. 4.88 days (P = 0.28).

Conclusion

ERAS protocol in patients undergoing lumbar spinal surgery incorporated the use of regional anesthesia via the mTLIP block, we observed there is a statistically significant reduction in the LOS for lumbar laminectomy and a significant reduction in opioid administration for lumbar laminectomies and spinal fusion surgery.

## Introduction

Enhanced recovery after surgery (ERAS) protocols apply a multidisciplinary approach to perioperative care to minimize the physiologic adverse effects that result from the stress of surgery [1,2]. Spinal fusion surgery is one of the most painful surgical procedures [3]. Many patients undergoing spinal surgery are opioid-tolerant, making postoperative pain difficult to control may need additional opioid medication in the perioperative period [4]. Many complex variables affect the postoperative course, with postsurgical factors playing a significant role [5, 6]. Recently, the ERAS Society has endorsed a protocol for lumbar spinal surgery in 2021 [7]. Although the ERAS protocols for spinal surgery are gaining popularity, at the time of our literature search, we found none that incorporated regional anesthesia.

In 2015, a regional technique targeting the lumbar area known as the thoracolumbar interfascial plane (TLIP) block was described by Hand et al. [8]. This block targets the dorsal rami of the lumbar spinal nerves. It is performed by injecting local anesthetic under ultrasound guidance in the fascial plane between the multifidus and longissimus muscles at the level of L2-L3 using a lateral to medial needle approach. Some have raised concerns over potential inadvertent intrathecal injection performing this block because of the proximity of the target fascial plane to the spinal cord and the lateral to medial needle approach. A modified approach to this block was described in 2017 [9]. This modified thoracolumbar interfascial plane (mTLIP) block also targets the dorsal rami of the lumbar spinal nerves; however, the fascial plane and needle approach are different. For this block, a local anesthetic is injected into the fascial plane between the iliocostalis and longissimus muscles using a medial to lateral needle approach. This theoretically decreases the chance of inadvertent intrathecal injection and provides a more consistent and reliable target on ultrasound imaging than the traditional approach.

For our protocol, we included traditional ERAS components, such as multimodal anesthesia, which has been shown to lead to a reduction in complications, length of stay (LOS), and hospital cost in patients undergoing spinal fusion surgery [2]. We incorporated analgesics, such as acetaminophen, gabapentin, and ketamine, which are frequently included in many ERAS protocols published by the ERAS Society. In addition, we added regional anesthesia, which generally has been shown to reduce pain, improve organ function, increase mobility, decrease nausea and vomiting, and reduce LOS when applied as part of an ERAS protocol [2]. Our objective was to analyze the ERAS protocol that incorporated regional anesthesia as a quality improvement (QI) measure to reduce the LOS and in-hospital opioid requirements.

## Materials and methods

**Figure 1:**
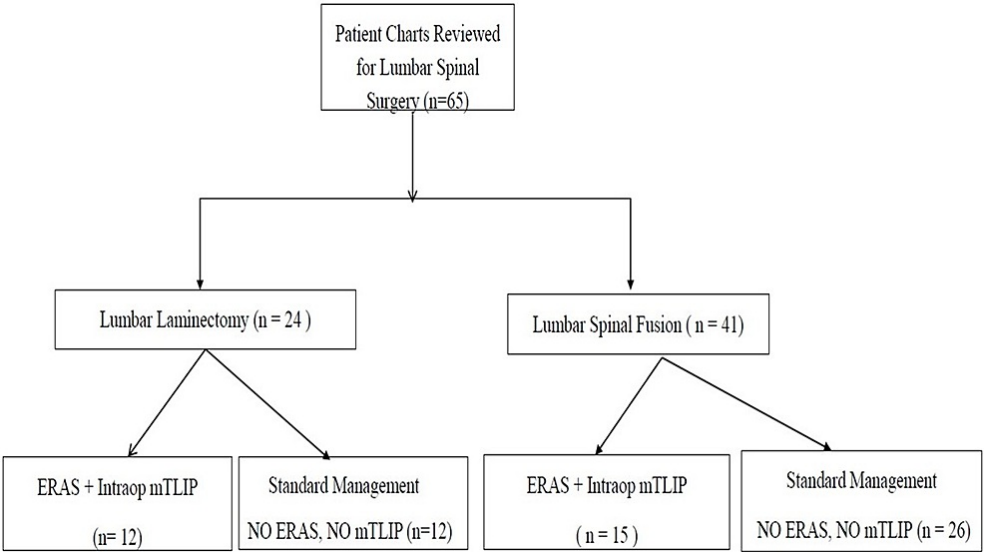
Flow diagram showing charts reviewed for ERAS quality improvement project. mTLIP: modified thoracolumbar interfascial plane; ERAS: enhanced recovery after surgery

This study was approved by the institutional review board. Data of patients undergoing lumbar spinal surgery from January 1, 2017, to August 31, 2018, without the ERAS protocol and data of patients undergoing lumbar spinal surgery from September 1, 2018, to November 30, 2019, using the ERAS protocol were collected. The ERAS QI protocol was implemented in collaboration between the Departments of Anesthesia and Neurosurgery. The patients included were adults aged > 18 years who underwent elective lumbar spinal fusion or lumbar laminectomy. The exclusion criteria were as follows: patients admitted for trauma, aged < 18 years, requiring dialysis, who refused to receive regional anesthesia, and allergic to protocol medications, non-English speaking patients, or pregnant patients (Figure 1).

The ERAS protocol was applied from September 2018 to November 2019, and data regarding total opioid administration and LOS were collected. A retrospective chart review examining similar data points was conducted for patients undergoing similar procedures but not included in the protocol in the previous 20 months. This information was used as a control for comparison. Multiple surgeons were included in this study. All surgeries were performed in the same hospital. The details of the protocol and evidence for their inclusion are listed below. A summary of the protocol is presented in Table 1.

**Table 1:** Summarized spinal surgery protocol *Preoperative - Upon arrival to preoperative holding area, PO- Oral, IV- Intravenous, PRN – as needed

Preoperative^*^	Intraoperative	Postoperative
Acetaminophen 1 gram PO	Standard induction and maintenance	Ketamine infusion (0.15 mg/kg/hour up to 72 hours)
Gabapentin 300 mg PO	Dexamethasone 8 mg (dose adjusted for diabetic pts)	Acetaminophen 650 mg PO every 6 hours scheduled
	Ketorolac 15 mg IV	Ketorolac 15 mg IV every 6 hours for 48 hours
	Ketamine 0.2 mg/kg IV bolus followed by 0.1 mg/kg/hour infusion	Methocarbamol 750 mg PO every 6 hours scheduled
	Tranexamic acid (10 mg/kg loading dose followed by 1 mg/kg/hour infusion)	Gabapentin 300 mg nightly scheduled
	Modified thoracolumbar interfascial plane block with liposomal bupivacaine	Oxycodone 10 mg PO every 4 hours PRN
		Hydromorphone 1 mg IV every 2 hours PRN

Acetaminophen

All patients received 1000 mg per os (PO) acetaminophen upon arrival to the preoperative holding area on the morning of surgery. Following the completion of the surgery, patients were administered scheduled doses of 650 mg every six hours while inpatient.

Gabapentin

All patients received 300 mg PO of gabapentin upon arrival to the preoperative holding area on the morning of surgery. Postoperatively, patients were administered scheduled doses of 300 mg every evening while in the hospital. If patients were previously taking gabapentin prior to surgery, their home dose was continued throughout the perioperative period.

Induction and maintenance of anesthesia

This was performed at the discretion of the attending anesthesia staff. When possible, the administration of opioids was avoided.

Dexamethasone

Non-diabetic patients received dexamethasone 8 mg intravenously (IV) after the induction of anesthesia and prior to surgical incision. Patients with diabetes were administered a reduced dose of 4 mg IV.

Ketamine

Patients were administered a 0.2 mg/kg of actual body weight IV bolus after the induction of anesthesia but prior to surgical incision. A maintenance dose of 0.1 mg/kg/hr IV was administered intraoperatively. A continuous infusion of 0.15 mg/kg/hr actual body weight was started upon arrival to the post-anesthesia care unit. This infusion was continued for up to 72 h and was stopped at the discretion of the primary surgical team.

Ketorolac

Patients were administered 15 mg IV of ketorolac after the induction of anesthesia but prior to surgical incision. Following surgery, patients were administered scheduled doses of 15 mg IV every 6 h for 48 h in patients with normal kidney function.

Tranexamic acid

After the induction of anesthesia, a bolus of 10 mg/kg of actual bodyweight of tranexamic acid was administered over 10 min. This was followed by an infusion of 1 mg/kg/h throughout the duration of surgery and was stopped when surgical closure began.

Regional anesthesia

**Figure 2:**
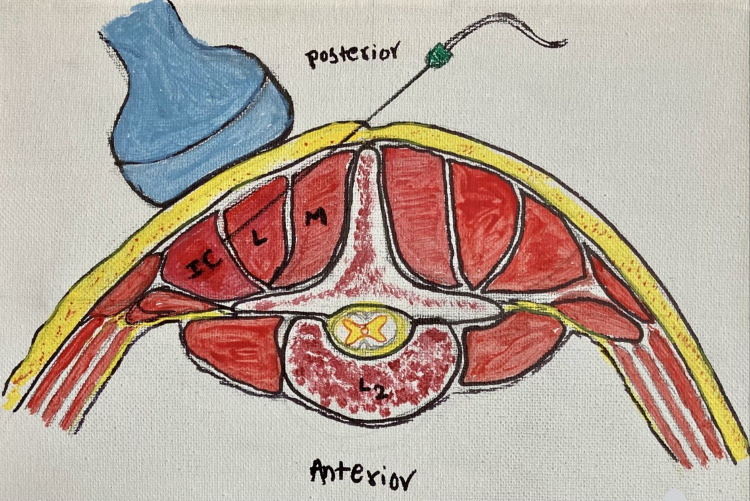
Modified thoracolumbar interfascial plane (mTLIP) block showing the tip of the needle location between the longissimus muscle and the Iliocostalis muscle at the L2 vertebral level. M- Multifidus, L- Longissimus, IC- Iliocostalis. Painting credit: Lakshmi N. Kurnutala, M.D., M.Sc.

Following the induction of anesthesia and prone positioning, a bilateral mTLIP block was performed. To accomplish this, the patient’s lumbar region was cleaned using chlorhexidine soap and then draped with sterile towels. Under ultrasound guidance, the spinous process of L2 was identified, and the probe was moved laterally until the fascial plane between the iliocostalis and longissimus muscles was identified. Subsequently, using the medial to lateral approach, a needle was inserted into the fascial plane, and 20 ml of dilute liposomal bupivacaine solution was injected. This process was repeated on the contralateral side. To prepare the dilute liposomal bupivacaine solution, a 20-ml vial of liposomal bupivacaine was added to 20 ml of sterile saline, resulting in 40 ml of solution. Half of the diluted solution was injected into each side (Figure 2).

Postoperative pain control

In addition to the scheduled acetaminophen, ketorolac, gabapentin, and ketamine infusion, patients were also administered scheduled methocarbamol 750 mg PO every 6 h, oxycodone 10 mg PO every 4 h as needed for moderate pain, and hydromorphone 1 mg IV every 2 h as needed for rescue analgesia.

Statistical analysis

For continuous variables, P values were computed using the Wilcoxon rank-sum test if normality checks using the Shapiro-Wilk test failed. Independent two-sample t-tests were used. If variances from pre-ERAS and post-ERAS protocols were found to be unequal using the F-test, then the Satterthwaite two-sample t-test was used. As this was a QI project and retrospective chart review, there was neither formal hypothesis testing nor formal sample size calculation.

## Results

Patient population: laminectomy

A total of 65 patients were included in the study. Twenty-four patients were included in the laminectomy arm in this study. Twelve patients received the ERAS protocol (treatment arm), and 12 underwent laminectomy without the ERAS protocol (control arm). Single-level and multilevel procedures were included (1-2/3 level). The mean ages of patients in the treatment and control arms were 57.4 and 56.6 years, respectively (Table 2). Similar distributions of men and women were observed in both the pre- and post-ERAS groups.

Patient population: spinal fusion

A total of 41 patients were included in the spinal fusion arm of this study. Fifteen patients received the ERAS protocol (treatment arm), and 26 patients underwent spinal fusion without the ERAS protocol (control arm). Single level, and multilevel procedures were included. The mean ages of patients in the treatment and control arms were 57.4 and 56.6 years, respectively. Similar distributions of men and women were observed in both the pre- and post-ERAS groups.

Clinical outcome measures

All patients in both arms of the study underwent surgery without any intraoperative events related to anesthesia. All patients tolerated the entire procedure. Postoperatively, pain control was successful with standard narcotic analgesic regimens. Comparison of opioid usage between pre- and post-ERAS for both study arms was performed after converting administered opioids to morphine milligram equivalent (MME). In addition, the LOS was also assessed for both arms of the study, and pre- and post-ERAS protocols were compared. The details of the MME and LOS for each procedure are listed below. There is no statistical difference between the two groups for preoperative opioids, 90-day readmissions, and perioperative morbidity.

Laminectomy

Mean opioid usage for the treatment group was 39.69 MME. This suggests a mean reduction in opioid use of 41.99 MME and represents a reduction in opioid requirements of 51% (P = 0.04). The mean LOS in the control arm was 2.79 days. In the treatment arm, the mean LOS was 0.75 days. This represents a mean reduction in LOS of 2.04 days or a 73% reduction (P = 0.01) (Table 2).

**Table 2:** Evaluation of length of stay (LOS), and opioids in patients undergoing laminectomy procedures pre- and post-enhanced recovery after surgery (ERAS) protocol Data are expressed as mean ± standard deviation. *P<0.05 significant

	Laminectomy (n=24)		
	Control group (Pre-ERAS ) (n=12)	Treatment group (ERAS) (n=12)	P^*^
LOS (days)	2.79 ± 1.71	0.75 ± 0.77^*^	0.01
Opioid equivalent (MME)	81.68 ± 44.07	39.68 ± 17.30^*^	0.03

Spinal Fusion

The mean opioid usage for the treatment arm was 97.12 MME. This represents a mean reduction in opioid use of 60.36 MME or 38%. The mean LOSs in the control and treatment arms were 4.88 and 5.4 days, respectively (P = 0.27) (Table 3).

**Table 3:** Evaluation of length of stay, and morphine milligram equivalent (MME) opioids in patients undergoing spinal fusion procedures pre- and post-enhanced recovery after surgery (ERAS) protocol. Data are expressed as mean ± standard deviation. *P<0.05 significant

	Spinal fusion (n=41)		
	Control group (Pre-ERAS) (n=26)	Treatment group (ERAS) (n=15)	P^*^
LOS (days)	4.88 ± 1.21	5.40 ± 1.284	0.28
Opioid equivalent (MME)	157.48 ± 43.49	97.12 ± 54.11^*^	0.04

## Discussion

Increasing emphasis is placed on reducing opioid use, improving patient outcomes, and maximizing hospital resources in patients undergoing spinal fusion surgery. Utilizing the ERAS protocols is one of the strategies performed to achieve these goals.

It has been shown that the application of the ERAS protocols for spinal surgery results in a reduction in opioid requirements and LOS. Most of the literature on spinal surgeries applying the ERAS protocols are retrospective [1,2,3,10] with only limited literature available on prospective studies [11,12].

We analyzed the use of the ERAS protocols with an intraoperative mTLIP block for lumbar spinal surgery. Regarding laminectomies, we were able to realize a statistically significant reduction in opioid administration and LOS. For lumbar fusion procedures, we did not observe any reduction in LOS; however, we observed a significant reduction in overall opioid administration. Debono et al.’s retrospective review of patients requiring anterior cervical discectomy and fusion, anterior lumbar interbody fusion, and posterior lumbar fusion compared LOS and postoperative complications before the implementation of the ERAS protocol and following the implementation of the ERAS protocol. Their review found that the ERAS protocol resulted in a significant decrease in LOS in all three groups without causing increased postoperative complications [10]. In another retrospective chart review, the authors examined the results of ERAS implementation for L1-L3 lumbar fusion. The ERAS cohort of 57 patients and a control cohort of 40 patients underwent similar procedures during the six months before the ERAS protocol implementation. In the ERAS group, the LOS was significantly shorter, and the postoperative requirement of oxycodone-acetaminophen was significantly less [13].

Wang et al. retrospectively compared 38 patients treated with the ERAS protocol to a group of 15 patients who underwent minimally invasive Transforaminal Lumbar Interbody Fusion (TLIF) prior to the introduction of the enhanced-recovery sequence. Differences between these groups included the use of endoscopic decompression, injections of liposomal bupivacaine, and the performance of surgery under sedation in the ERAS group. Shorter LOS was observed in the ERAS group [14]

Brady wood et al. [15] and Sivaganesan et al. [16] in their retrospective review, found that the ERAS protocol for lumbar spinal surgery has significantly decreased LOS. A review by Smith et al. comprised patients who underwent L1-L2 lumbar fusion. A comparison of 96 patients who received the ERAS protocol with 123 patients who underwent the same surgical procedure prior to the initiation of the ERAS protocol (no ERAS protocol) found a significant decrease in postoperative opioid and rescue antiemetic use, but interestingly there was no significant difference in postoperative pain scores or LOS between the two groups [17].

As the ERAS protocol has become more popular and been widely used, there are a couple of recent prospective studies shedding light on the ERAS protocol and its role and scope. Soffin et al.’s prospective randomized controlled trial assessing the quality of patient recovery tested the effect of an enhanced recovery pathway on the quality of recovery after L1-L2 lumbar fusion. In this, researchers found significantly higher Quality of Recovery-40 scores in the enhanced recovery group on the third postoperative day [12]. Though the statistically significant gains in early recovery were achieved using an enhanced recovery pathway, the study failed to demonstrate a significant clinical effect in the ERAS group. The authors are not sure which pathway elements or combinations of elements helped to improve the quality of recovery. The results support the potential for enhanced recovery pathways to optimize recovery after lumbar spinal fusion [12].

A prospective randomized study of adult patients undergoing spinal surgery by Maheshwari et al. studied the effect of using a combination of four nonopioid analgesics versus placebo on the postoperative opioid consumption, pain scores, and quality of recovery [11]. In this study authors found that using an analgesic pathway based on preoperative acetaminophen and gabapentin, combined with intraoperative infusions of lidocaine and ketamine, did not improve recovery in patients who underwent multilevel spinal surgery [11]. Although the individual role and contribution of the regional anesthesia in the ERAS protocol need to be further defined, the literature has consistently reported that supporting regional, such as epidural catheter [18,19] and ON-Q Pain Buster pump (B. Braun, Melsungen, Germany) [20], significantly reduce pain and intraoperative and postoperative use of opioids.

Our study is the first review to analyze the ERAS protocol, which incorporates regional anesthesia as a component. This review analyzed the use of the ERAS protocol with an intraoperative mTLIP block (a measure to reduce the LOS and in-hospital opioid requirements). We observed a statistically significant reduction in overall opioid administration in laminectomies and lumbar fusion procedures. However, a decrease in the LOS was observed only in laminectomies. We hypothesize that the reduction in opioid requirements observed in our study could be largely due to the mTLIP regional technique used. This is an area of future study.

Our study has some limitations. First, our sample size was limited, and both ERAS and control groups were retrospectively examined. As mentioned above, regional anesthesia was used in conjunction with multiple other treatment elements, making it unclear what role each individual treatment contributed to the outcome. Over the past several years, laminectomy surgery has observed a global reduction in LOS, with many of these procedures being performed in an outpatient setting. Whether our results regarding lumbar laminectomy simply mirror the national trend or are an outcome of our intervention remains unclear. The prospective randomized control studies of ERAS protocols for lumbar spine surgery with and without regional anesthesia (mTLIP block) may give a better idea in the future to assess the benefit of regional anesthesia.

## Conclusions

In conclusion, in patients treated with the ERAS protocol undergoing lumbar spinal surgery with the use of regional anesthesia via the mTLIP, we observed a statistically significant reduction in opioid administration and LOS for laminectomies. In patients undergoing spinal fusion surgery with the ERAS protocol with the use of regional anesthesia via the mTLIP, there is a statistically significant reduction in opioid administration, but no reduction in LOS was observed. 
